# Recent progress in performance optimization of Cu-SSZ-13 catalyst for selective catalytic reduction of NO_*x*_

**DOI:** 10.3389/fchem.2022.1033255

**Published:** 2022-10-17

**Authors:** Pan Li, Ying Xin, Hanxue Zhang, Fuzhen Yang, Ahui Tang, Dongxu Han, Junxiu Jia, Jin Wang, Zhenguo Li, Zhaoliang Zhang

**Affiliations:** ^1^ School of Chemistry and Chemical Engineering, Shandong Provincial Key Laboratory of Fluorine Chemistry and Chemical Materials, University of Jinan, Jinan, China; ^2^ National Engineering Laboratory for Mobile Source Emission Control Technology, China Automotive Technology & Research Center Co., Ltd., Tianjin, China

**Keywords:** Cu-SSZ-13 catalyst, selective catalytic reduction, low-temperature activity, hydrothermal stability, poisoning resistance

## Abstract

Nitrogen oxides (NO_*x*_), which are the major gaseous pollutants emitted by mobile sources, especially diesel engines, contribute to many environmental issues and harm human health. Selective catalytic reduction of NO_*x*_ with NH_3_ (NH_3_-SCR) is proved to be one of the most efficient techniques for reducing NO_*x*_ emission. Recently, Cu-SSZ-13 catalyst has been recognized as a promising candidate for NH_3_-SCR catalyst for reducing diesel engine NO_*x*_ emissions due to its wide active temperature window and excellent hydrothermal stability. Despite being commercialized as an advanced selective catalytic reduction catalyst, Cu-SSZ-13 catalyst still confronts the challenges of low-temperature activity and hydrothermal aging to meet the increasing demands on catalytic performance and lifetime. Therefore, numerous studies have been dedicated to the improvement of NH_3_-SCR performance for Cu-SSZ-13 catalyst. In this review, the recent progress in NH_3_-SCR performance optimization of Cu-SSZ-13 catalysts is summarized following three aspects: 1) modifying the Cu active sites; 2) introducing the heteroatoms or metal oxides; 3) regulating the morphology. Meanwhile, future perspectives and opportunities of Cu-SSZ-13 catalysts in reducing diesel engine NO_*x*_ emissions are discussed.

## Graphical Abstract

Table of Contents.
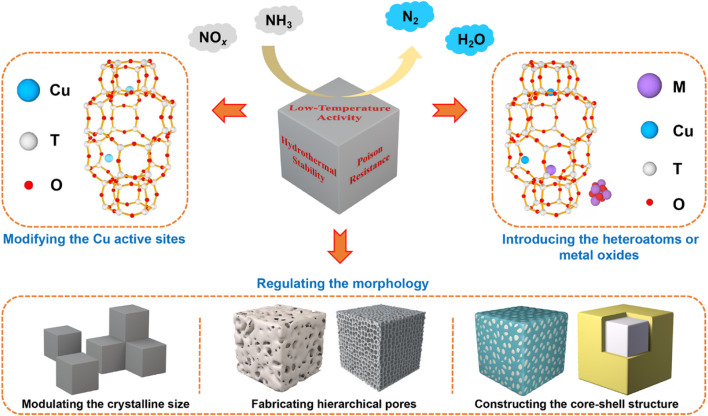

## 1 Introduction

Nitrogen oxides (NO_*x*_) are the major gaseous pollutants, which contribute to the problems of air pollution, playing roles in the formation of acid rain, photochemical smog, haze, and ozone (Richter et al., 2005). Nowadays, diesel exhaust becomes the primary and most fundamental source of NO_*x*_ emission as the increasing number of diesel engines, which are more powerful and fuel-efficient than similar-sized gasoline engines (Wu et al., 2023). Therefore, controlling the NO_*x*_ emission from diesel engines is urgent and imperative to meet current and future emission regulations such as China 6, U.S. EPA Tier 3, and Euro 7 (Yang et al., 2017; Barbosa, 2020). Among the various after-treatment technologies, the selective catalytic reduction (SCR) of NO_*x*_ with NH_3_/urea in oxygen-rich exhausts has been regarded as the most effective method to reduce NO_*x*_, in which it converts NO_*x*_ into N_2_ and H_2_O with the aid of a catalyst (Guan et al., 2014; Isabella and Enrico, 2014). However, the application of commercialized vanadia-based SCR catalyst (V_2_O_5_-WO_3_/TiO_2_) was restrained by the latest emission regulations due to the insufficient low-temperature activity and thermal stability as well as the toxicity of V_2_O_5_ (Xu et al., 2021). Metal-exchanged zeolite catalyst especially copper (Cu)-exchanged SSZ-13 zeolite (Cu-SSZ-13) was developed and industrially applied to substitute for V_2_O_5_-WO_3_/TiO_2_ catalyst on diesel vehicles, due to its high activity and hydrothermal stability (Kwak et al., 2010; Gao et al., 2013a; Beale et al., 2015; Paolucci et al., 2016a; Wang et al., 2017; Xin et al., 2018; Zhang S. T. et al., 2020; Pu et al., 2020).

Indeed, along with the application of Cu-SSZ-13 catalyst in diesel engine SCR systems, some practical challenges emerged owing to the highly dynamic conditions encountered in diesel engine emissions (Mohan et al., 2020). As in idling or cold start condition, the diesel exhaust temperature is much lower than the light-off temperature of the Cu-SSZ-13 catalyst. Thus, the first challenge is to further improve the catalytic conversion of NO_*x*_ at low temperatures by decreasing the light-off temperature of the Cu-SSZ-13 catalyst (Pu et al., 2020). The typical operating temperature range of SCR catalyst under normal conditions is from 200 to 300°C, while the exhaust temperature can shoot to 600°C due to the thermal regeneration of diesel particulate filter upstream of the catalyst. Together with water composition in diesel engine exhaust, the aluminum (Al) sites in the Cu-SSZ-13 framework were attacked leading to dealumination, and the migration and aggregation of active Cu species, resulting in the deactivation of the catalyst (Zheng et al., 2020; Lyu et al., 2021; Simancas et al., 2021). Therefore, another challenge is that Cu-SSZ-13 catalyst is required to have adequate high-temperature hydrothermal stability. In addition to the low-temperature activity and hydrothermal stability, the poisoning resistance to water, sulfur, and hydrocarbons (fuel-derived contaminants), phosphorous and zinc (derived from lubricating oil additives), alkali metals (potassium, calcium, sodium, and magnesium, originating from urea solution and detergent additives), are also great challenges for the practical application of Cu-SSZ-13 catalysts on diesel vehicles (Lezcano-Gonzalez et al., 2014; Beale et al., 2015; Fan et al., 2018a; Guo et al., 2021).

**FIGURE 1:**
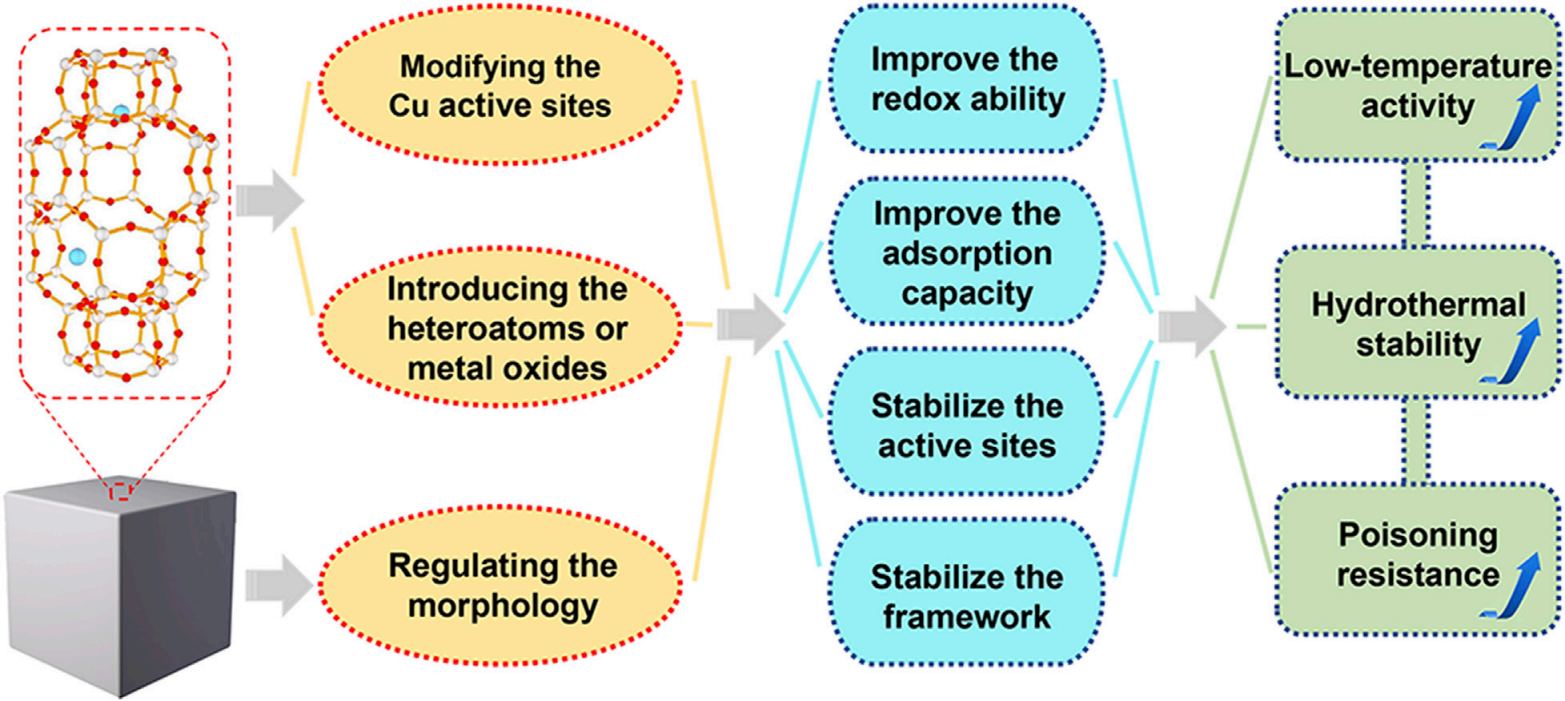
The optimization strategies and corresponding principles of Cu-SSZ-13 catalysts for NH_3_-SCR reaction.

To overcome the above-mentioned challenges of Cu-SSZ-13 catalyst and achieve high-efficient NO_*x*_ removal, more and more researchers have focused on the improvement of low-temperature activity, hydrothermal stability, and poisoning resistance. Generally, all these catalytic performances could be modified by the synthesis methods, Cu active sites, and structures of Cu-SSZ-13 catalyst. In this review, we summarize the recent progress in optimizing the SCR performance of Cu-SSZ-13 and discuss these researches according to the following three aspects: 1) modifying the Cu active sites; 2) introducing the heteroatoms or promoters; 3) regulating the morphology (Figure 1). Last but not the least, we present some of the future perspectives and opportunities of Cu-SSZ-13 catalysts in the field of NO_*x*_ emission control, hoping to make some guiding suggestions for the following research.

## 2 Modifying the Cu active sites

**FIGURE 2:**
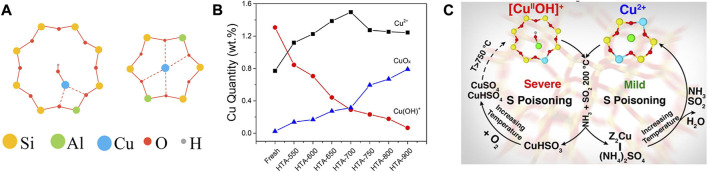
**(A)** The schematic representation of (Left) the 8 MR with a single Al atom and a Cu(OH)^+^ complex in CHA cage and (Right) the 6 MR with two Al atoms located diagonally and a Cu^2+^ cation in the center. **(B)** Estimation of Cu^2+^, Cu(OH)^+^, and CuO_x_ in fresh and hydrothermally aged Cu-SSZ-13 catalysts. Reproduced with permission Song et al. (2017), Copyright 2017, American Chemical Society. **(C)** The SO_2_ poisoning mechanism of Cu^2+^ and Cu(OH)^+^ sites. Reproduced with permission Jangjou et al. (2018), Copyright 2018, American Chemical Society.

As is widely accepted, Cu^2+^ ions and CuO_*x*_ are the predominant Cu species in Cu-SSZ-13 catalyst, especially the former is thought to be the active sites for SCR reaction, and the latter is proved to be active for the undesirable NH_3_ oxidation (Fickel and Lobo, 2010; Korhonen et al., 2011; Deka et al., 2012; Gao et al., 2014; Shan et al., 2021). Along with the deep investigation, the active Cu^2+^ ions were found in two locations in Cu-SSZ-13 as shown in Figure 2A, thereof the relatively stable Cu^2+^ ions coordinated with two framework Al atoms (Cu^2+^-2Z, Z_2_Cu) are isolated at the windows of six-membered rings (6 MR) and [Cu(OH)]^+^ species coordinated with one framework Al atom ([Cu(OH)]^+^-Z, ZCuOH) appear and populate inside the CHA cages next to eight-membered rings (8 MR) (Kwak et al., 2012b; Andersen et al., 2014; Paolucci et al., 2016b). Although both of the Cu^2+^ ions are active for SCR reaction, they exhibit unique reactivity and stability due to their different coordination environments (Gao et al., 2017; Paolucci et al., 2017; Zhang et al., 2022). At low temperature, ZCuOH is estimated to be 1.5 times more active for SCR than Z_2_Cu, therefore increasing the population of ZCuOH sites appears to benefit low-temperature SCR activity (Song et al., 2017). Simultaneously, Z_2_Cu is proved to possess higher hydrothermal stability than ZCuOH. With increasing aging severity, ZCuOH gradually converts to CuO_*x*_ clusters that accelerate the collapse of the zeolite framework, resulting in the hydrothermal deactivation of the Cu-SSZ-13 catalyst (Figure 2B). Further studies provide strong evidence to demonstrate that ZCuOH is much more vulnerable than Z_2_Cu to poisoning (Figure 2C) (Luo et al., 2016; Jangjou et al., 2018; Tang et al., 2021). Accordingly, the amount, type, and location of Cu species are closely related to the catalytic performance of Cu-SSZ-13, which could be modified by the synthesis conditions and elemental compositions (Clemens et al., 2015; Han M.-J. et al., 2018; Villamaina et al., 2019; Zhang J. et al., 2020).

### 2.1 Synthesis methods

The synthesis methods strongly affect the nature and location of Cu species in zeolites, which directly determines the NH_3_-SCR activity (Zhang et al., 2016b; Han M.-J. et al., 2018). The general synthesis strategies of Cu-SSZ-13 have been reviewed in previous work, amongst the conventional aqueous ion-exchange (IE), solid-state ion exchange (SSIE) and one-pot synthesis are the most commonly used methods to obtain Cu-SSZ-13 catalysts (Xin et al., 2018; Zhang L. et al., 2019). Basically, the varieties and ratio of reactants, nature of parent zeolites, as well as processing parameters of IE (such as pH value, IE temperature and time, *etc.*) are critical issues in the preparation of Cu-SSZ-13. The effects of synthesis methods as well as these critical factors on the physicochemical properties and catalytic performance of Cu-SSZ-13 catalysts will be described in detail below.

#### 2.1.1 Aqueous ion-exchange method

**FIGURE 3:**
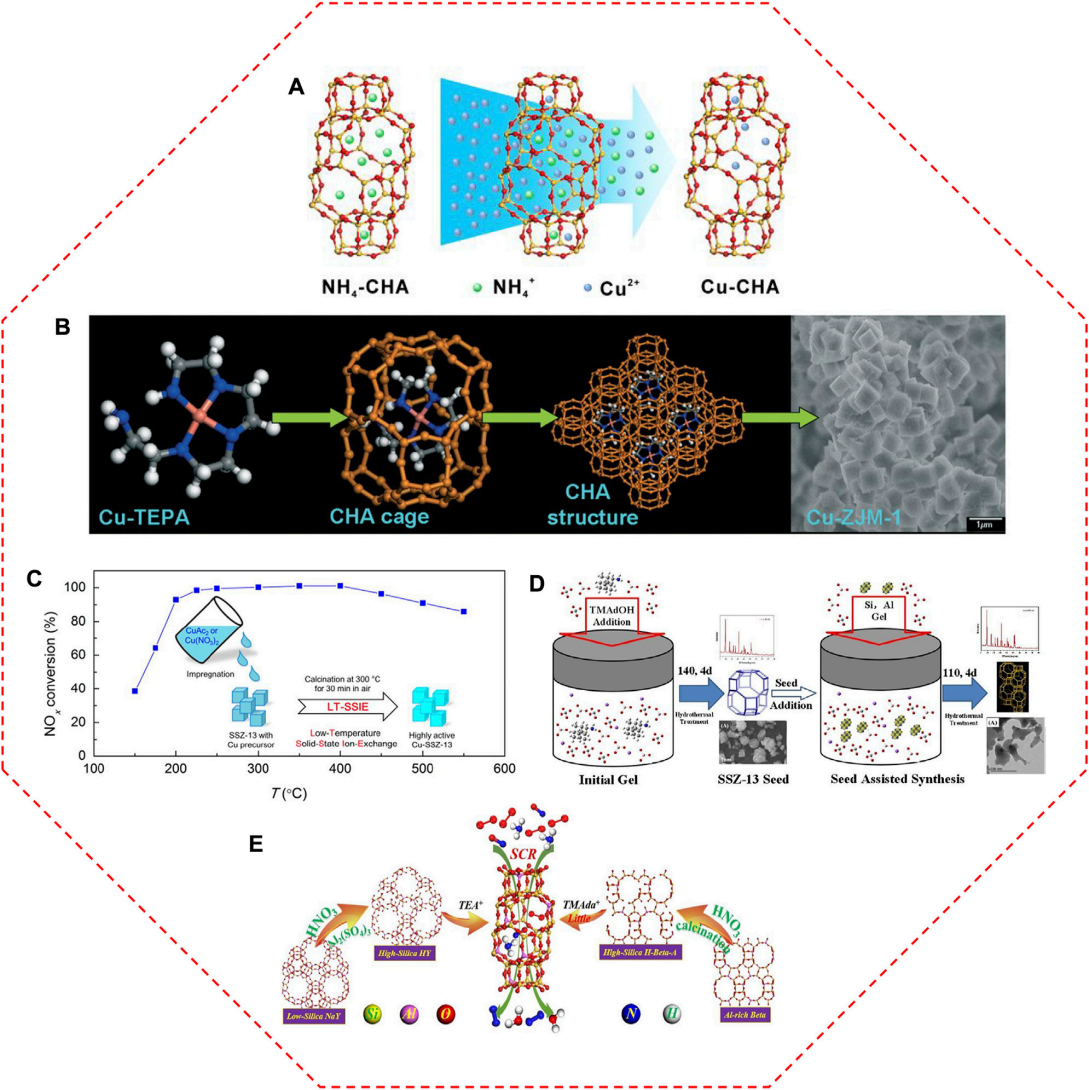
The representative synthesis methods for Cu-SSZ-13 catalysts. **(A)** Schematic diagram of aqueous ion exchange method. Reproduced with permission Xin et al. (2018), Copyright 2018, John Wiley and Sons. **(B)** Mechanism of one-pot synthesis method with Cu-TEPA template. Reproduced with permission Ren et al. (2011), Copyright 2011, Royal Society of Chemistry. **(C)** Diagram of low-temperature solid-state ion-exchange method and corresponding NO_x_ conversion of the obtained Cu-SSZ-13 catalyst. Reproduced with permission Ma et al. (2019), Copyright 2019, American Chemical Society. **(D)** Synthetic process of seed-oriented synthesis method. Reproduced with permission Zhang X. Y. et al. (2019), Copyright 2019, Elsevier Publishers. **(E)** The inter-zeolite conversion route with low-cost NaY and Al-rich beta precursors and alternately low cost or low content structure directing agents. Reproduced with permission Zhang et al. (2021), Copyright 2021, Elsevier Publishers.

Traditionally, Cu-SSZ-13 catalyst is prepared *via* the aqueous IE of NH_4_/SSZ-13 with Cu salts (Figure 3A), in which the Cu^2+^ ions are atomically dispersed by anchoring on the IE sites owing to the compensation for the positive charge of the zeolite framework (Deka et al., 2013; Xin et al., 2018; Zhang L. et al., 2019; Shan et al., 2021). Based on this principle, the Cu loading, as well as the type of Cu species in Cu-SSZ-13, can be readily modified by changing the aqueous solution concentration (Gao et al., 2013b; Wang X. F. et al., 2022). Kwak et al. (2012a) controlled the IE levels of Cu-SSZ-13 from 20% to 80% by increasing the amount of Cu^2+^ ions in the Cu(NO_3_)_2_ solution, and the SCR activity was correspondingly improved at low temperatures. With the increase of Cu concentration (0–0.125 mol/L, CuCl_2_ aqueous solutions), the SCR performance of Cu-SSZ-13 catalysts increases initially and decreases subsequently in the low-temperature region (Zhou et al., 2021). When the concentration of the Cu salt solution reaches up to 0.100 mol/L, the obtained Cu-SSZ-13 catalyst exhibits the highest low-temperature SCR activity. The excessive Cu^2+^ ions replaced the H^+^ ions in the Brønsted acid sites, inhibiting the storage and transfer capacity of NH_3_.

The low-temperature SCR performance of Cu-SSZ-13 catalysts was also affected by using different copper salts as precursors, and the light-off temperature followed the order of copper acetate (CuAc_2_) < copper chloride (CuCl_2_) < copper sulfate (CuSO_4_) < copper nitrate (Cu(NO_3_)_2_) (Zhou et al., 2022). That is because CuAc_2_ endows the Cu/SSZ-13 catalyst with the largest number of isolated Cu^2+^ and strong Lewis acid sites, facilitating the low-temperature SCR reaction. Basically, the content and location of the Cu^2+^ ions and CuO species in the Cu-SSZ-13 catalysts could be adjusted by Cu precursors (Wang X. F. et al., 2022). CuCl_2_ and CuAc_2_ enabled most of the Cu^2+^ ions located at 6MRs, while that located at 8 MR were prepared using Cu(NO_3_)_2_ and CuSO_4_. Therefore, relatively high stability of Cu-SSZ-13 was obtained by using CuCl_2_ and CuAc_2_ as precursors during the IE process, even though Cu(NO_3_)_2_ and CuSO_4_ precursors provided an unexpected increase of the high-temperature activity after hydrothermal treatment due to the accelerated adsorption of NO_*x*_ species.

Furthermore, the high-performance Cu-SSZ-13 could be directly obtained from Na-SSZ-13 by adjusting the pH values of Cu salt solution during the ion-exchange process (Zhao H. W. et al., 2021). In the absence of H^+^ ions, the Cu^2+^ ions only exchanged the Na^+^ ions in 8 MR as ZCuOH sites due to the steric hindrance of Na^+^ ions located in 6MRs, while as H^+^ ions were present, they competed with Cu^2+^ ions to exchange the Na^+^ sites. Therefore, the Cu^2+^ ions took a priority over H^+^ ions to occupy the neighboring Al sites in 6MRs, causing that Z_2_Cu dominated in Cu-SSZ-13. The sufficient H^+^ induced a slight deactivation for Cu-SSZ-13 after hydrothermal aging owing to the fact that the stable Z_2_Cu restricted the formation of undesired CuAl_2_O_4_ and ultimately improved the hydrothermal stability.

#### 2.1.2 One-pot synthesis

To reduce the number of manufacturing steps and complexity as well as to enhance the synthesis efficiency, Cu species were directly introduced into the channels of SSZ-13 zeolites during the crystallization process (Ren et al., 2012; Martínez-Franco et al., 2013; Wang J. et al., 2021). Ren et al. (2011) and Ren et al. (2012) rationally designed an efficient one-pot synthesis of Cu-SSZ-13 catalysts, in which the high Cu loading and high dispersion of Cu species were simultaneously achieved using the low-cost Cu-tetraethylenepentamine (Cu-TEPA) complex as the sole template instead of the traditional template (*N,N,N*-trimethyl-1-adamantammonium hydroxide, TMAdaOH) (Figure 3B). Compared with the conventional IE method, the one-pot route introducing Cu^2+^ species into the zeolites greatly enhanced the efficiency of Cu species, inducing good SCR activity, especially in the low-temperature range. A large amount of isolated Cu^2+^ sites in the one-pot synthesized Cu-SSZ-13 catalyst account for the stronger Lewis acid sites and more active nitrate species, and thus providing the superior low-temperature activity (Zhang et al., 2016b). However, the Cu-SSZ-13 catalyst derived from the one-pot synthesis method suffers from too excessive Cu content, which is harmful to its hydrothermal stability (Xie et al., 2014). The loss of a majority of nitrate adsorbed sites and acid sites led to the lower SCR performance of Cu/SSZ-13 catalysts after hydrothermal aging, while the counterpart prepared by the IE method exhibited higher low-temperature efficiency due to its suitable content of isolated Cu^2+^ sites and applicable proportion of Z_2_Cu/ZCuOH (Jiang H. et al., 2020). The effects of Cu precursors on the Cu active sites were also detected in the Cu-SSZ-13 catalysts derived from one-pot synthesis method (Wang M. X. et al., 2019). The NO_*x*_ conversion of Cu-SSZ-13 catalyst prepared by Cu(NO_3_)_2_ precursor reached 90% at 180°C and remained above 90% at a wide temperature range of 180–700°C. After hydrothermal aging at 800°C, it still exhibited above 90% NO conversion under a temperature range of 240–600°C. In comparison with CuSO_4_ and CuCl_2_ precursors, the smallest Pauling radium of NO_3_^−^ in Cu(NO_3_)_2_ precursor facilitated the entering of Cu^2+^ ions into the pores of SSZ-13 to generate more isolated Cu^2+^ sites and relatively uniform distribution, thus providing the outstanding low-temperature SCR activity. In addition, based on Hofmeister anion effects, the strongest adsorption capacity of NO_3_^−^ among the three kinds of anions (NO_3_^−^, Cl^−^, and SO_4_^2−^) is conducive to the formation and growth of crystal grain, thereof contributing to the superior anti-aging properties.

To overcome the drawbacks of the one-pot synthesis method, a new one-pot method of Cu-SSZ-13 zeolites was developed by the cooperative utilization of the Cu-TEPA complex and organic *N,N,N*-trimethyl-1-adamantammonium (TMAda) as organic structure-directing agents (OSDAs) (Martínez-Franco et al., 2013). The specific combined templates enable the direct introduction of metal extra-framework species in controlled amounts and thus guarantee the excellent catalytic activities and hydrothermal stabilities of Cu-SSZ-13. Alternatively, the excessive Cu loading of one-pot synthesized Cu-SSZ-13 catalyst can be lowered by a “reverse” IE with NH_4_NO_3_ solution (Guo et al., 2014; Xie et al., 2014). This post-treatment procedure removed a part of Cu^2+^ ions, and simultaneously relocalized the reserved ones from the large cages to 6 MR sites to prevent the formation of bulk CuO, which enhanced the SCR activity and the hydrothermal stability of the Cu-SSZ-13 catalyst. The post-treatment with HNO_3_ (Xie et al., 2015; Jiang H. et al., 2020; Liu B. et al., 2022) and HNO_3_-NH_4_NO_3_ (Shan et al., 2020) could also optimize the Cu species distribution and avoid the aggregation of isolated Cu^2+^ ions into CuO_*x*_ clusters to achieve a wide active temperature window, high hydrothermal stability, and enhanced anti-poisoning performance of Cu-SSZ-13 catalyst. Moreover, the post-treatment with ammonium hexafluorosilicate (AHFS) guaranteed the improved hydrothermal stability of Cu-SSZ-13 catalyst due to the inhibition of dealumination and structural collapse and the formation of Si(OSi)_3_(OAl) and Si(OSi)_4_ structures with better hydrothermal stability (Zhang et al., 2015). Meanwhile, the OH^−^ ions of the surface Si−OH groups could be exchanged with F^−^ ions of the decomposed AHFS to form a highly stable hydrophobic surface to avoid the water erosion of the Cu-SSZ-13 catalyst.

Besides the aforementioned post-treatment, thermal aging without water and mild hydrothermal aging (<700°C) could induce the migration of metastable ZCuOH to unsaturated 6 MR sites to form Z_2_Cu (Zhang et al., 2020c; Ma et al., 2020; Lyu et al., 2021). Since the SCR reaction kinetics is governed by the diffusion of Cu^2+^ ions, the Cu^2+^ ion mobility determines the activity of the Cu-SSZ-13 catalyst. Based on this correlation and the migration of Cu^2+^ ions during hydrothermal aging, Lee et al. (2021b) rationally controlled the ratio of Cu species (ZCuOH and Z_2_Cu) with different ion mobilities in Cu-SSZ-13 catalyst by treating under a hydrothermal condition (under 20% O_2_, 10% H_2_O balanced with N_2_ at 550°C) for various time. With increasing the aging time, the proportion of ZCuOH decreased leading to the declined low-temperature activity, and Cu/SSZ-13 catalyst with a higher proportion of ZCuOH demonstrated superior low-temperature reactivity. In addition, the ZCuOH sites are more susceptible to sulfur poisoning and interacted more readily with SO_2_ to form the highly stable bisulfite/bisulfate species, resulting in the deactivation (Jangjou et al., 2018). Therefore, mild hydrothermal aging at 650°C could also enhance the sulfur poisoning resistance (Xi et al., 2021).

Nevertheless, the effects of severe hydrothermal aging on the SCR performance of Cu-SSZ-13 catalyst remains controversial. The common idea is that ZCuOH aggregates at >700°C, and two types of Cu species (CuO_*x*_ and CuAlO_*x*_) are formed during hydrothermal aging, which is detrimental to the SCR activity and selectivity (Ma et al., 2020). On the contrary, hydrothermal aging was demonstrated to facilitate the conversion from CuO to Cu^2+^, resulting in the preservation of sufficient isolated Cu^2+^ ions for maintaining high NH_3_-SCR reaction activity at low temperatures (Lyu et al., 2021).

#### 2.1.3 Solid-state ion-exchange method

The solid-state ion exchange (SSIE) method is another successful choice for the synthesis of Cu-SSZ-13 catalyst, therein the zeolites are adequately mixed with Cu-containing chemicals, and the obtained mixture is heated to high temperatures to proceed with the IE procedure (Wang et al., 2014). Compared with the aqueous IE method, the SSIE method is relatively simple, rather straightforward, and controllable of Cu loading. However, some drawbacks impeded its application, such as partial damage to the zeolitic structure, the incomplete reaction of Cu precursor as well as very high reaction temperatures (Xin et al., 2018). Actually, the required temperature for the Cu^2+^ exchanges during SSIE can be lowered in the presence of NH_3_ or a standard NH_3_-SCR feed owing to the NH_3_-solvated Cu^2+^ ions ([Cu(NH_3_)_2_]^+^) intermediates with high mobility facilitating the SSIE process (Shwan et al., 2014; Clemens et al., 2015). With the aid of a thermally unstable Cu salt (CuAc_2_ or Cu(NO_3_)_2_) as the precursor, the high-performance Cu-SSZ-13 catalyst was obtained by a low-temperature SSIE method (Figure 3C) (Ma et al., 2019).

#### 2.1.4 Improved synthesis methods

Although Cu species are crucial to SCR performance, the support, SSZ-13 is also a vitally important consideration for the application of Cu-SSZ-13 catalysts. The high crystallinity and specific surface area as well as the regular morphology of SSZ-13 are beneficial to the well-dispersion of active sites and structural stability, guaranteeing the enhanced SCR activity of the Cu-SSZ-13 catalyst (Wang X. H. et al., 2020; Jabri et al., 2020). To enhance the synthesis efficiency and improve SCR performance of Cu-SSZ-13, some efficient and low-cost strategies have been developed, such as using cheaper OSDA (Chen et al., 2014), adding seeds (Wang et al., 2018; Zhang X. Y. et al., 2019; Zhao et al., 2019b), interzeolite conversion from FAU zeolites (Nedyalkova et al., 2013; Xiong et al., 2017; Lv et al., 2022), and microwave-assisted synthesis (Han L. et al., 2018; Wang B. et al., 2021).

Zhang and colleagues developed an economical way for SSZ-13 preparation with the essentially cheap choline chloride as a template (Chen et al., 2014; Wang X. H. et al., 2020). Under optimization by the fluoride-assisted synthesis, the obtained SSZ-13 catalyst exhibited excellent SCR performance with 100% NO conversion from 150 to 550°C due to the developed pore structure, strong NH_3_ adsorption capacity, and low CuO content (Wang X. H. et al., 2020). To increase the yield of SSZ-13, researchers reported a solvent-free method by using *N,N,N*-dimethylethylcyclohexylammonium bromide (DMCHABr) as a template (Shan et al., 2019; Zhang J. et al., 2020). Remarkably, the obtained Cu-SSZ-13 possessed more Z_2_Cu, thus showing higher hydrothermal stability compared with that synthesized by the traditional method using TMAdaOH as a template.

Seed-assisted method can reduce crystallization time, increase crystallinity, and adjust the particle size in the final product (Zhang J. et al., 2020). An organotemplate-free synthesis of SSZ-13 was also developed *via* the utilization of seeds (Zhao et al., 2017; Zhang X. Y. et al., 2019; Wang Y. et al., 2020). This seed-guided method is following a “core-shell” growth mechanism, in which the Si source and Al source continue to grow on the seed during the crystallization process (Figure 3D) (Zhang X. Y. et al., 2019). Cu-SSZ-13 derived from the seed-assisted method has a high amount of isolated Cu^2+^ ions in 6 MR due to a high fraction of paired Al sites, leading to a high ability for resisting the harsh hydrothermal aging (Wang Y. et al., 2020). Even though the particle size could be decreased by increasing the seed amounts, the turnover frequencies (TOFs) of the catalysts with different particle sizes and Cu loadings were identical, due to their similar Si/Al ratio, acidity, and Cu distributions (Wang et al., 2018). SAPO-34 micro-crystallite was also an ideal candidate for seed to synthesize the SSZ-13 crystal, which enhanced the Al distribution and influenced the type and stability of the isolated Cu^2+^ ions, as well as largely prevented the agglomeration of isolated Cu^2+^ ions, thus generating improved hydrothermal stability (Zhao et al., 2019b).

Furthermore, the rapid synthesis of SSZ-13 through interzeolite transformation from FAU zeolite was achieved (Nedyalkova et al., 2013; Kim et al., 2014; Xiong et al., 2017). The interzeolite conversion method could generate more paired Al atoms occupied 6 MR to provide stronger hydrothermally stable sites and better high-temperature SCR performance (Lv et al., 2021a). Zhang et al. (2021) further refined the interzeolite conversion method by using the low-cost NaY and Al-rich beta precursors (Figure 3E). After ion exchange with Cu, the Cu-SSZ-13 catalyst showed superior deNO_*x*_ performance compared with that prepared by the conventional method, which can be attributed to its high crystallinity and surface area as well as abundant acidic sites.

SSZ-13 zeolites can also be synthesized with a short crystallization time assisted with microwave treatment to achieve excellent particle dispersion and a regular morphology (Han L. et al., 2018; Wang B. et al., 2021). Compared to the traditional hydrothermal method, the microwave hydrothermal method has the advantages of fast and uniform heating, fast reaction rate, and low energy consumption (Han L. et al., 2018). The corresponding Cu-SSZ-13 possessed a large specific surface area and abundant Z_2_Cu ions, leading to excellent catalytic activity. Moreover, the microwave treatment strengthened the Al−O−Si bonds to stabilize the zeolite framework, thereby gaining excellent hydrothermal stability.

To sum up, the aqueous IE method enables most of the Cu^2+^ ions locate at IE sites and few excessive Cu^2+^ ions disperse on the surface of SSZ-13 zeolite. Although this method could avoid the formation of undesired CuO_*x*_, the loading amount of Cu^2+^ is heavily dependent on the properties of SSZ-13 zeolite (such as Si/Al ratio and crystallinity), which is the core limitation for acquiring more Cu^2+^ active sites. By contrast, the one-pot synthesis method could introduce more Cu^2+^ active sites into the zeolites with high efficiency and less complexity, however the enhanced low-temperature SCR activity of the obtained Cu-SSZ-13 catalyst is usually at the expense of its hydrothermal stability. Fortunately, this drawback could be overcome by using specific combined templates or post-treatment. Currently, the SSIE method is the most straightforward way to synthesize Cu-SSZ-13 with controllable Cu loading, but the structural damage of the zeolite and the suitable Cu precursor are still problems for attention. Fundamentally, the above-mentioned methods as well as the improved synthesis methods are based on the excellent parent zeolite, thus it is crucial to synthesize SSZ-13 zeolite with high crystallinity, specific surface area, and stability. The combination of the outstanding SSZ-13 and synthesis method is a major challenge to achieve high-performance Cu-SSZ-13 catalysts with satisfactory hydrothermal stability and poisoning resistance.

### 2.2 Zeolite framework composition

In addition to the synthesis methods, the amount and type of Cu species in Cu-SSZ-13 are complicated by the zeolite framework composition such as Si/Al ratio (Fan et al., 2018b) and Al distribution (Zhang J. et al., 2020; Chen Z. X. et al., 2022). Moreover, the relative populations of Z_2_Cu and ZCuOH are dependent on these factors (Paolucci et al., 2016b). Therefore, tuning the zeolite framework composition is crucial for the Cu-SSZ-13 catalyst as it establishes the interaction between Cu redox sites and residual Brønsted acid sites which determine the catalytic performance during the SCR reaction.

#### 2.2.1 Si/Al ratio

**FIGURE 4:**
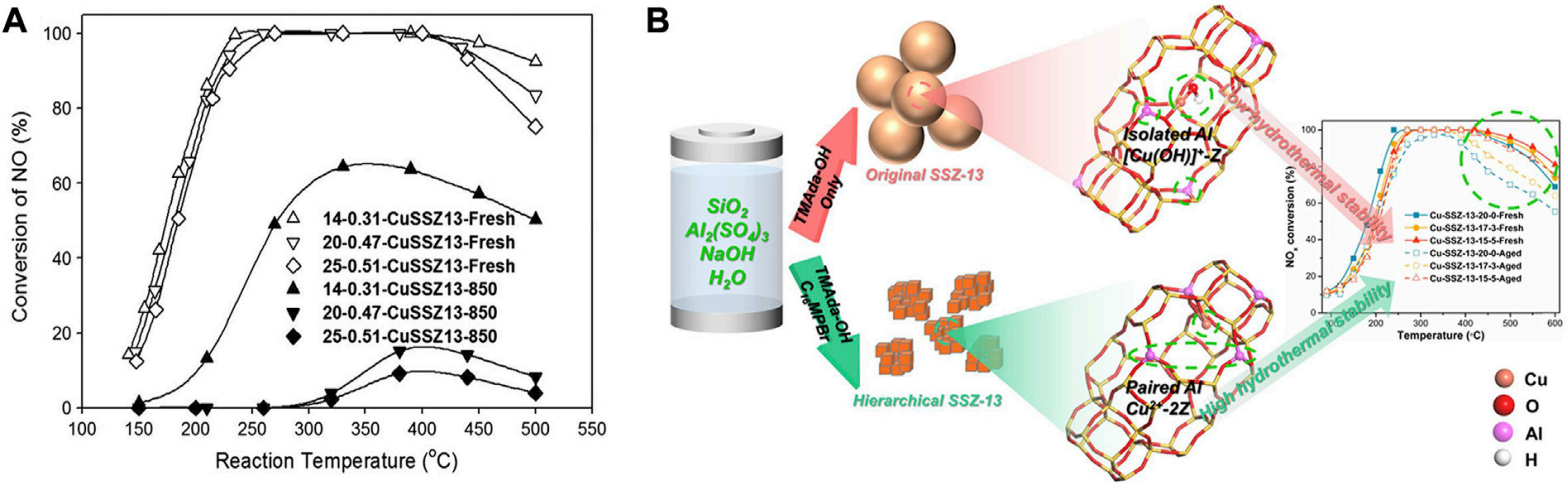
The effects of zeolite framework composition of Cu-SSZ-13 catalysts on Cu^2+^ active sites and SCR performance. **(A)** The deNO_x_ performance of Cu-SSZ-13 catalysts with different Si/Al ratios before and after hydrothermal aging at 850°C. Reproduced with permission Kim et al. (2014), Copyright 2014, Elsevier Publishers. **(B)** Collaboration of TMAda-OH and C_16_MPBr templates induced more paired Al and Z_2_Cu sites contributing to the improved hydrothermal stability. Reproduced with permission Chen Z. X. et al. (2022), Copyright 2022, Elsevier Publishers.

As shown in Figure 4A, the Si/Al ratio is vital for the SCR performance of the Cu-SSZ-13 catalyst (Kim et al., 2014; Martini et al., 2022). The high concentration of Si in zeolite ensures the framework’s stability, while the substitution of Si by Al supplies acid sites owing to the charge compensation, which further increases the number of active Cu^2+^ ions during the IE process and improves the NH_3_ adsorption during SCR reaction (Gao et al., 2015b; Fan et al., 2018b). In addition, the nature of the multiple Cu species as well as the Cu^2+^ ions locations and redox properties could be systemically tuned by the Si/Al ratio of the Cu/SSZ-13 catalyst, which significantly affects the activity and stability (Gao et al., 2015b; Martini et al., 2022).

The low Si/Al ratio ensures the high Brønsted acidity density and large Cu ion-exchange capacity of Cu-SSZ-13, which are beneficial to improving the SCR activity (Paolucci et al., 2016b). Cu-SSZ-13 (Si/Al = 6) was proved to have superior SCR activity compared with those with a high Si/Al ratio (12 or 35) and the equivalent Cu loading (Gao et al., 2015b). Especially, the low Si/Al ratio could introduce more Cu species to generate more reactive Z_2_Cu sites to compose the paired isolated Cu^+^ species during SCR reaction, which accelerated the oxidation half-cycle of the redox cycling and thus promoted the low-temperature activity (Zhang et al., 2020d; Liu et al., 2022b). However, the large number of Al and Cu active sites are detrimental to the hydrothermal stability of Cu-SSZ-13. With increasing the Si/Al ratio, the number of highly stable Cu^2+^ ions (Z_2_Cu) decreases, which is attributed to the less Al content in Cu-SSZ-13 catalyst, resulting in the decreased activity of Cu-SSZ-13 catalyst (Han et al., 2017; Fan et al., 2018b; Jiang et al., 2019). Meanwhile, the unstable isolated Cu^2+^ ions (ZCuOH) transformed into the aggregated CuO species during the hydrothermal aging treatment, resulting in the collapse of the zeolite structure and giving rise to the deactivation of the Cu-SSZ-13 catalyst.

Only the Cu-SSZ-13 catalyst with an appropriate Si/Al ratio could possess both excellent low-temperature activity, hydrothermal stability, and poisoning resistance (Fan et al., 2018b). Feng et al. (2020) compared the NH_3_-SCR performance of a series of Cu-SSZ-13 catalysts with different Si/Al molar ratios (Si/Al = 10.6, 13.0, or 16.0), therein Cu-SSZ-13 with Si/Al ratio of 13.0 exhibited the best NH_3_-SCR performance, especially the low-temperature activity, due to the highest BET surface area, redox capacity and surface acidity. The too high Si/Al ratio (Si/Al > 25) decreased the number of active sites and lacks sufficient acidity resulting in inferior activity over the whole temperature range (Liu et al., 2022b). Therefore, by controlling the Si/Al ratio, the content of active sites could be regulated and the obtained catalysts would possess better low-temperature performance or higher hydrothermal stability.

#### 2.2.2 Al distribution

Although the amount and type of Cu species in Cu-SSZ-13 are significantly affected by Si/Al ratios, the coordination structure and location of the Cu species as well as acid site distribution, which directly affect the SCR performance and hydrothermal stability, are fundamentally determined by the Al distribution in Cu-SSZ-13 zeolites (Shan et al., 2021). Density functional theory (DFT) calculations combined with *ab initio* molecular dynamics simulations confirmed that the Al distribution markedly affected the formation of Cu(NH_3_)^2+^-pair during SCR reaction, and uncovered a low-energy and entropically preferred path for O_2_ activation and dissociation over Cu(NH_3_)^2+^-pair (Chen L. et al., 2018). The results suggested the low-temperature SCR activity for Cu-SSZ-13 was governed by the formation of Cu-pairs, indicating that precise synthesis of Cu-SSZ-13 concerning Al distribution may enhance the catalytic activity.

Recently, it has been demonstrated that the Al distribution in the framework of SSZ-13 can be controlled by changing the starting materials in the synthesis procedure (Iorio Di and Gounder, 2016; Nishitoba et al., 2018). The Al distribution at a fixed Si/Al ratio could be adjusted by using selected structure-directing agents (SDAs) to synthesize the SSZ-13 zeolite (Iorio et al., 2020). Zhang J. et al. (2020) reported that framework Al distribution can be optimized by using *N,N*-dimethylcyclohexylammonium (DMCHA^+^) as a template to increase the proportion of close Al sites so that more Z_2_Cu can be stabilized at 6 MR sites against hydrothermal aging. C_16_H_33_-[N^+^-methylpiperidine] (C_16_MPBr) was also used to collaborate with TMAdaOH to introduce the hydrophobic mesoporous system into the Cu-SSZ-13 catalyst. The increasing amount of C_16_MPBr induced the increased density of paired Al in the SSZ-13 framework to promote the generation of more stable Z_2_Cu species, which effectively suppressed the loss of active Cu^2+^ ions and dealumination of the framework during the hydrothermal aging (Figure 4B) (Chen Z. X. et al., 2022). The organic template-free synthesis method could also be applied to obtain Cu-SSZ-13 catalyst with a high amount of isolated Cu^2+^ ions in 6 MR (Wang Y. J. et al., 2020). Therein, the high fraction of paired Al sites in the catalyst stabilized Cu^2+^ ions in the 6MRs, leading to a high ability to resist the harsh hydrothermal aging. Furthermore, Cu-SSZ-13 zeolite prepared by the F^−^-aided template-free synthesis method possessed a high fraction of paired Al and active Z_2_Cu species and thereby exhibits good catalytic performance and hydrothermal stability in SCR reaction (Lv et al., 2021a).

Using SAPO-34 as the seed in the hydrothermal synthesis of Cu-SSZ-13 facilitates the coordination of Si with (−O−Al), making a high concentration of Al distribution and isolated Z_2_Cu sites (Zhao et al., 2019b). The SAPO-34 seed moderated the dealumination as well as the agglomeration of the active Cu^2+^ ions in the Cu-SSZ-13 in the hydrothermal working condition, thus providing enhanced hydrothermal stability of the catalyst. Moreover, the Al distributions and Cu^2+^ locations could be regulated by using different types of alkali metal cations (Li^+^, Na^+^, K^+^, and Cs^+^) as counter-cations in SSZ-13 zeolites (Lv et al., 2021b). The paired Al species and distorted AlO_4_^−^ tetrahedra increased with decreasing radii of alkali metal cations, which led to an increase of isolated Cu^2+^ species and the enhanced SCR performance of the Cu-SSZ-13 catalyst. Additionally, phosphorus (P) had also been used to modulate the Al sites in the Cu-SSZ-13 catalyst with a low Si/Al ratio, in which P ions were coordinated with the framework-bonded Al species to form a framework silicoaluminophosphate interface, retarding the dealumination of Cu-SSZ-13 catalyst and thus improving the hydrothermal stability significantly (Zhao et al., 2019a).

### 2.3 Cu content or Cu/Al ratio

**FIGURE 5:**
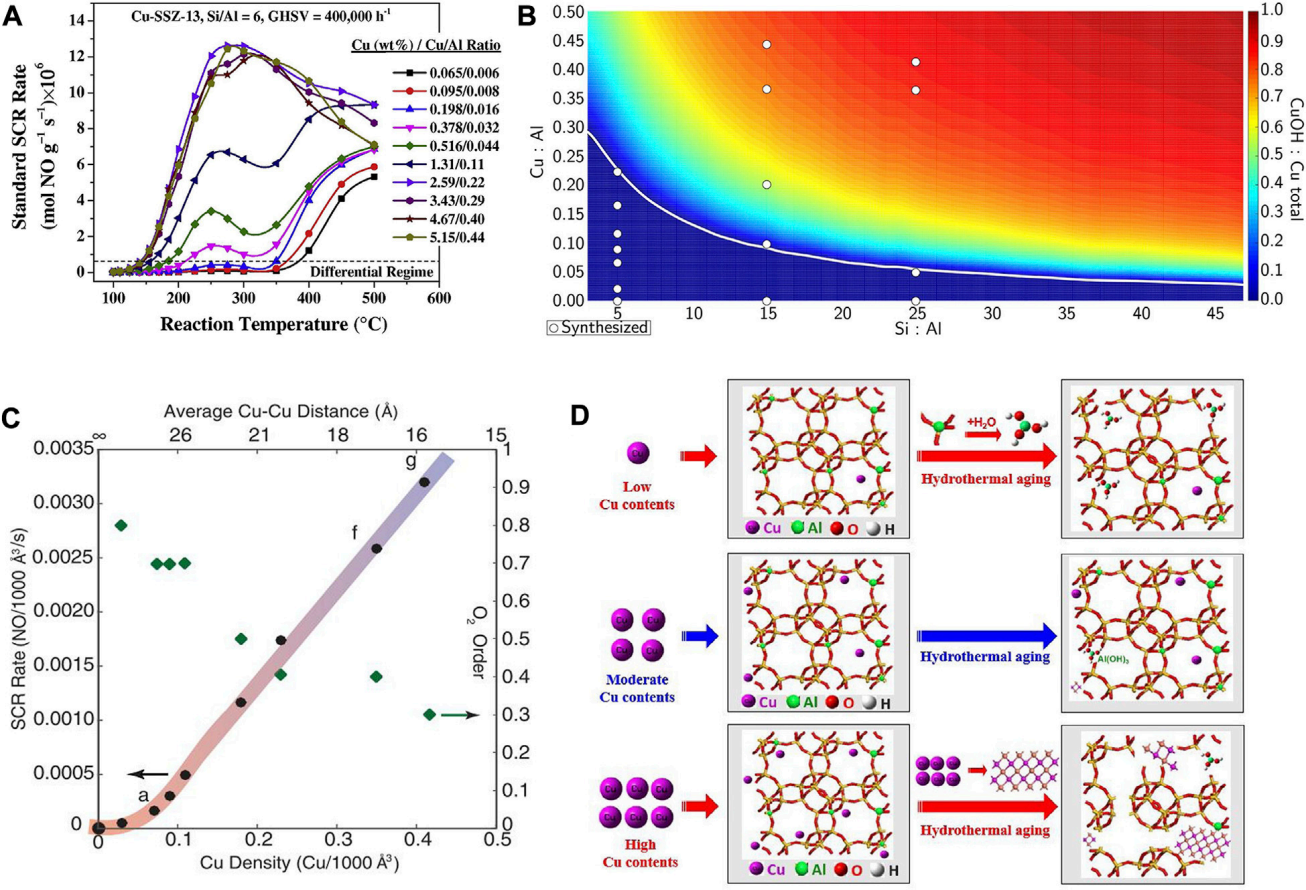
The effect of Cu content or Cu/Al ratio on the active sites and SCR performance of Cu-SSZ-13 catalysts. **(A)** Standard SCR rates as a function of temperature for Cu-SSZ-13 catalysts with various Cu-loadings and Cu/Al ratios. Reproduced with permission Gao et al. (2014), Copyright 2014, Elsevier Publishers. **(B)** Predicted Cu site compositional phase diagram *versus* Si:Al and Cu:Al ratios. Reproduced with permission Paolucci et al. (2016b), Copyright 2016, American Chemical Society. **(C)** Standard NO_x_ SCR rates (per volume catalyst; 473 K; measured in a differential reactor by using a gas mixture representative of practical low-temperature application, including 2.5% H_2_O) and apparent O_2_ orders measured on Cu-CHA-X samples (Si/Al = 15) of increasing Cu ion density. Reproduced with permission Paolucci et al. (2017), Copyright 2017, American Association for the Advancement of Science. **(D)** The deactivation mechanism of hydrothermal aging of Cu-SSZ-13 with different Cu contents. Reproduced with permission Shan et al. (2020), Copyright 2020, Elsevier Publishers.

The Cu contents as well as Cu/Al ratio of the Cu-SSZ-13 catalyst are also key factors influencing its SCR performance because their variation could change the distribution of ZCuOH and Z_2_Cu sites in the zeolite (Figures 5A,B) (Gao et al., 2014; Paolucci et al., 2016b; Villamaina et al., 2019). Zhou et al. (2021) demonstrated that the SCR performance of the Cu-SSZ-13 (Si/Al ≈ 22) catalysts increases initially and decreases subsequently with an increase in Cu contents in the low-temperature region (100–300°C). This result could be rationalized by the variation of amount of isolated Cu^2+^ species and different acid sites, therein the amount of Lewis acidity and isolated Cu^2+^ species were increased as the Cu contents increases from 0.07 to 0.21 mmol/g, while most of the H^+^ in the Brønsted acid sites (Si−OH−Al) are replaced by the further increased isolated Cu^2+^. Consequently, the storage and migration of NH_3_ were inhibited to reduce the rate of SCR activity. Under the condition of the same Si/Al ratio, the Cu/SSZ-13 catalyst with a higher Cu/Al ratio had better low-temperature performance because most of the active sites are in the form of ZCuOH (Liu et al., 2022c). Instead, when Cu/Al ratio is relatively low, the active sites are more in the form of Z_2_Cu, so as to the hydrothermal stability and poisoning resistance of the Cu-SSZ-13 catalyst are enhanced. Paolucci et al. (2017), Verma et al. (2014) demonstrated the high Cu/Al ratio is ideal for SCR reaction due to the preferred form of dimeric Cu sites, which are the active sites for reaction temperatures <250°C, leading to high SCR activity at low temperatures (Figure 5C). However, at high temperatures (>350°C) these moieties become thermally stable, occupy the CHA cage and obstruct pore openings, thus resulting in decreased NO_*x*_ reduction efficiency (Gao et al., 2014).

Furthermore, by changing the Cu/Al ratio, the type, location, and proportion of Cu^2+^ active sites as well as the SCR performance can be effectively controlled (Chen J. W. et al., 2018). Shan et al. found that by reducing the Cu/Al ratios *via* HNO_3_ and NH_4_NO_3_ solution treatments, the hydrothermal stability of Cu/SSZ-13 catalyst could be effectively promoted (Figure 5D) (Shan et al., 2020). Experimental and calculation results demonstrated that the SCR reaction rate increased linearly with Cu/Al ratio up to Cu/Al = 0.2 for Cu-SSZ-13 catalyst with Si/Al = 4.5, and the effective maximum amount of isolated Cu occupying 2Al on the 6 MR of the SSZ-13 was achieved, contributing to the high activity and stability (Bates et al., 2014). With the increasing Cu/Al ratio, the overall intrinsic activity and hydrothermal stability of Cu-SSZ-13 catalysts decreased due to the linearly increased proportion of ZCuOH/Z_2_Cu (Kim et al., 2014). Cu-SSZ-13 catalyst with Cu/Al_2_O_3_ = 2.50 (Si/Al = 10; Cu content: 3.25 wt%) exhibits excellent catalytic activity (operation temperature window of 145–490°C) and adaptability to high gas hourly space velocity as well as hydrothermal stability (Chen J. W. et al., 2018). This could be ascribed to the presence of different functional Cu^2+^ species located in various sites in the catalyst. Some Cu^2+^ species situated close to 8 MR play a critical role in the low-temperature activity, while the others located in the center of the hexagonal prism are the most stable active species and simultaneously contribute to the excellent hydrothermal aging and SO_2_ resistance.

## 3 Introducing the heteroatoms or metal oxides

Besides the modification of the Cu active sites, introducing the heteroatom or promoter is also an efficient way to improve the performance of Cu-SSZ-13 catalysts. According to the previous studies, the active sites of Cu-SSZ-13 could be identified by characterization and performance testing, such as X-ray absorption fine structure, electron paramagnetic resonance, nuclear magnetic resonance, H_2_ temperature-programmed reduction, NH_3_ temperature-programmed desorption, diffuse reflection using Fourier transform spectroscopy, kinetic tests, and so on (Fickel and Lobo, 2010; Korhonen et al., 2011; Deka et al., 2012; Gao et al., 2014; Zheng et al., 2020; Shan et al., 2021; Wang Z. H. et al., 2022). By using these characterization methods, Cu^2+^ ions are still proved to be active sites after introducing heteroatom or promoter in Cu-SSZ-13 catalyst. The improved SCR performance is due to the increased number of active sites, and enhanced acidity and redox property of the catalyst, which are derived from the interaction between Cu^2+^ and heteroatom/promoter (Zheng et al., 2020; Wang Z. H. et al., 2022). Moreover, the introduced heteroatom or promoter could stabilize the active sites and the framework of zeolite, and improve the dispersion of active sites to provide favorable hydrothermal stability and anti-poisoning performance (Xie et al., 2022).

### 3.1 Alkali metal ions

**FIGURE 6:**
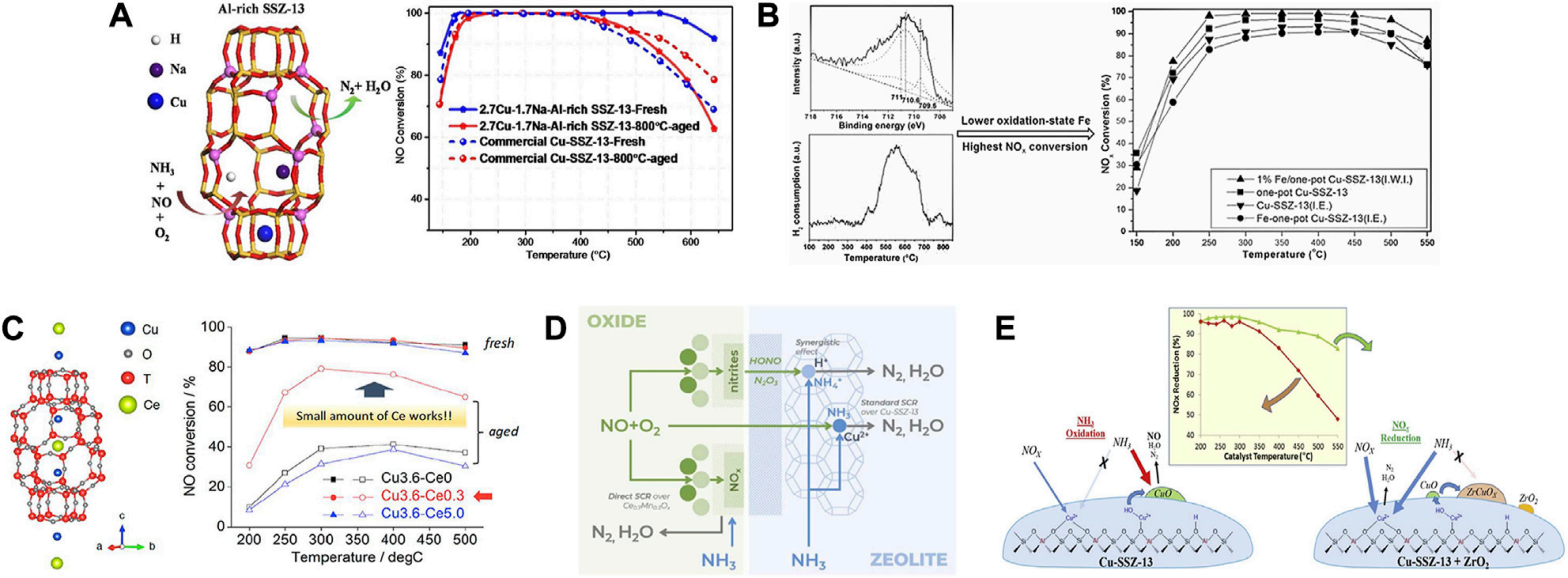
**(A)** Effects of Na^+^ ions on the activity and hydrothermal stability of Cu-SSZ-13 catalysts. Reproduced with permission Zhao et al. (2017), Copyright 2017, Elsevier Publishers. **(B)** Effects of Fe impregnation on the activity of Cu-SSZ-13 catalyst. Reproduced with permission Chen M. Y. et al. (2021), Copyright 2016, Royal Society of Chemistry. **(C)** Location of the Cu and Ce cations in Cu-SSZ-13 and the effect of Ce loading on the SCR performance and hydrothermal stability. Reproduced with permission Usui et al. (2018), Copyright 2018, American Chemical Society. **(D)** Schematic diagram of SCR mechanism of MnO_x_-CeO_2_-Cu-SSZ-13 composite catalyst. Reproduced with permission Andana et al. (2022), Copyright 2022, Elsevier Publishers. **(E)** Enhanced high-temperature SCR activity and schematic diagram of the highly dispersed ZrO_2_ modified Cu-SSZ-13. Reproduced with permission Peng et al. (2020), Copyright 2020, Elsevier Publishers.

Table 1 summarized the currently developed Cu-SSZ-13 catalysts doped with alkali metal ions, which will be discussed in detail below. Alkali metal ions, especially Na^+^ ions, are generally introduced in the synthesis of SSZ-13 zeolites by the hydrothermal method. As the common co-cations in Cu-SSZ-13 zeolites, alkali metal ions often influence the properties of active species and the zeolite support (Fan et al., 2018a; Wang C. et al., 2020). Certain amount of alkali metal ions could stabilize the Al-rich Cu-SSZ-13 catalysts and weaken the interaction between Cu and SSZ-13 framework to improve the reducibility, showing the improved SCR activity (Figure 6A) (Gao et al., 2015a; Xie et al., 2015; Zhao et al., 2017). Ulteriorly, the introduced alkali metals could improve the SCR performance of Cu-SSZ-13 catalysts along with decreasing radii of alkali metal cations (Li^+^, Na^+^, K^+^, and Cs^+^) owing to the increased paired Al species and distorted AlO_4_^−^ tetrahedra (Lv et al., 2021b). It is worth noting that Na^+^ ions could provide extra low-temperature NH_3_ storage to promote the low-temperature NO_*x*_ conversion, while the excess Na^+^ ions, competed with Cu^2+^ ions resulting in the depletion in the numbers of active Cu^2+^ sites (Gao et al., 2015a).

**TABLE 1:** Summary of the Cu-SSZ-13 catalysts doped with alkali metal ions.

Introduced species	Cu content (wt%)	Doped ions	Dopant content (wt%)	Active temperature window for fresh samples (°C) (NO_*x*_ conversion >90%)	Active temperature window for hydrothermal aged samples (°C) (NO_*x*_ conversion >90%)	References
Alkali metal ions	1.78	Li	0.03	225–500	325–475	Lv et al. (2021b)
	0.98	Li	0.4	200–500	225–500	Gao et al. (2015b)
	2.7	Na	1.7	150–650	200–600	Zhao et al. (2017)
	1.69	Na	0.07	250–500	225–475	Lv et al. (2021b)
	0.98	Na	1.78	180–500	225–500	Gao et al. (2015a)
	1.0	Na	1.5	210–500	--	Cui et al. (2020)
	1.73	Na	0.7	300–400	--	Chen et al. (2020c)
	1.46	K	0.11	250–500	250–500	Lv et al. (2021b)
	0.98	K	4.21	225–500	--	Gao et al. (2015a)
	1.50	Cs	0.76	250–500	--	Lv et al. (2021b)

However, the effect of alkali ions on the hydrothermal stability of Al-rich Cu-SSZ-13 catalyst is still under debate. For the one-pot synthesized Cu-SSZ-13 catalyst, the excessive residual Na^+^ ions after post-treatment with dilute HNO_3_ were detrimental to the hydrothermal stability because they decreased the stability of Cu species seriously (Xie et al., 2015). While for the ion-exchanged one, the introduced alkali metal cations (Na^+^ and Li^+^) helped decrease the excess Brønsted acidity and mitigated the hydrolysis of zeolite during hydrothermal aging to enhance the hydrothermal stability of Cu-SSZ-13 catalyst (Gao et al., 2015a; Cui et al., 2020).

To better differentiate the introduced Na^+^ ions interacted with active Cu^2+^ sites, the parent SSZ-13 zeolites of Cu-SSZ-13 catalysts with identical Si/Al ratio and Na loading were prepared by *in situ* hydrothermal synthesis, post-ion-exchange, and impregnation methods (Chen Z. X. et al., 2020; 2019). The post-ion-exchanged Na^+^ ions located on the 6 MR sites enriched the hydrothermally vulnerable ZCuOH species in Cu-SSZ-13 catalyst, while the impregnated Na^+^ ions reduced the number of Cu^2+^ ions significantly by forming CuO_*x*_ species and Cu-aluminate-like species to impede the oxidation half cycle of Cu^2+^, which was the rate-limiting step of SCR reaction at low temperatures on Cu/SSZ-13 catalyst. Surprisingly, the residual Na^+^ ions in situ hydrothermal synthesis exhibited only a negligible effect on the active sites and promoted the hydrothermal stability of Cu-SSZ-13 by preserving Si–OH–Al bonds, consequently maintaining the high SCR activities even after hydrothermal aging.

### 3.2 Transition metal ions

Introducing an appropriate amount of transition metal element into Cu-SSZ-13 is another method to improve the SCR performance and hydrothermal stability of catalysts. As shown in Table 2, transition metals, such as Fe, Mn, and Co are often used to modify the catalytic activity of Cu-SSZ-13 catalysts as popular active components (Zhang S. T. et al., 2020). Among these, Mn or Fe modified Cu-SSZ-13 catalysts have been extensively studied because of their excellent SCR performance.

**TABLE 2:** Summary of the Cu-SSZ-13 catalysts doped with transition metal ions.

Introduced species	Cu content (wt%)	Doped ions	Dopant content (wt%)	Active temperature window for fresh samples (°C) (NO_*x*_ conversion >90%)	Active temperature window for hydrothermal aged samples (°C) (NO_*x*_ conversion >90%)	References
Transition metal ions	3.99	Mn	1.68	200–450	300–400	Wang et al. (2022c)
	0.20 (IE degree)	Mn	0.10 (IE degree)	175–525	200–500	Song et al. (2019)
	2.6 (at%)	Mn	1.8 (at%)	140–330	--	Wan et al. (2021)
	2.71	Fe	0.357	200–450	200–375	Yang et al. (2012)
	1.50	Fe	0.63	160–580	210–550	Wan et al. (2021)
	4.51	Fe	5.1	150–450	--	Wang et al. (2022b)
	4.67	Fe	5.37	175–450	200–350	Zhang et al. (2014)
	8.0	Fe	1.0	250–500	250–500	Yin et al. (2016)
	2.3	Fe	0.89	200–550	250–350	Chen et al. (2021a)
	2.55	Fe	1.49	200–500	200–500	Wu et al. (2022)
	4.8	Fe	0.5	200–550	200–400	Yue et al. (2020)
	3.3	Fe	3.0	250–450	--	Wang et al. (2019b)
	0.99	Co.	0.82	300–450	300–450	Lee et al. (2021a)
	2.0	Nb	0.7	200–625	--	Wang et al. (2021b)
	2.4	Zn	8.3	250–600	300–550	Xu et al. (2020)

Mn was impregnated into Cu-SSZ-13 and MnO_*x*_ species tended to be located in the pores of Cu-SSZ-13 in a highly dispersed form, and markedly facilitated the redox ability of Cu-SSZ-13 (Du et al., 2020). The improvement of the redox ability promoted the formation of nitrate species, which were critical intermediates in the NH_3_-SCR process. As a result, the low-temperature catalytic performance was greatly improved in Mn-impregnated Cu-SSZ-13 catalysts. Furthermore, the low-temperature SCR performance could be also improved by the promoted oxidation of Cu^+^ to Cu^2+^ relying on the introduction of Mn (Jiang L. et al., 2020). By using co-exchange method, a series of Cu-Mn-SSZ-13 catalysts were obtained, and the moderate amount of Mn in the Cu-Mn-SSZ-13 catalyst significantly increased the SCR activity at the low-temperature range and reinforced the resistance to hydrothermal aging because Mn inhibited the aggregation of Cu species and the pore destruction of the catalyst (Song et al., 2019). Recently, MnCu-SSZ-13 catalysts with hierarchical pores were successfully prepared by a one-pot method using the complex templates, and the obtained catalyst exhibited excellent SCR activity and hydrothermal stability owing to the highly dispersed Mn species and the unique NO adsorption properties (Wang Z. H. et al., 2022). Particularly, the hierarchical pores structure of MnCu-SSZ-13 played key roles in the distribution of active sites and gas diffusion.

Yang et al. (2012) compared the SCR performance by incorporating a secondary metal cation M (Sc^3+^, Fe^3+^, In^3+^, and La^3+^) in the Cu-exchanged SSZ-13 catalyst, and the CuFe-SSZ-13 catalyst offered the best NO_*x*_ conversion activity in the 150–650°C range among the reported catalysts here. The introduced Fe^3+^ ions were in the vicinity of Cu^2+^ ions, and the heterobimetallic core facilitated the disproportionation reaction between NO and NO_2_ to form and stabilize NO^+^ and NO_2_^−^, thus executing the fast SCR reaction to contribute to a superior low-temperature SCR activity. Normally, the introduction of Fe^3+^ ions can significantly improve the high-temperature activity of Cu-SSZ-13 since Fe-SSZ-13 exhibits high NO_*x*_ conversion at higher temperatures in SCR reaction. In principle, the isolated Cu^2+^ species act as primary active sites for the low-temperature SCR reaction, while monomeric Fe^3+^ species provide sufficient active sites to sustain the SCR activity at high temperatures (Wan et al., 2021). According to this principle, incorporating these two elements into SSZ-13 by IE method could overcome the single metal deficiency and construct a more efficient catalyst for NH_3_-SCR reaction over a wide temperature range (Zhang et al., 2014; Yin et al., 2016; Wang X. Y. et al., 2022). The superior performance with a Fe/Cu molar ratio of 1.29 was attributed to the collaboration of various active species, including [Cu(OH)]^+^, [Fe(OH)]^2+^, and oligomeric iron, in which the former accounted for the excellent low temperature (<200°C) performance, while the later ones as well as isolated Cu^2+^ were responsible for the high-temperature activity (Wang X. Y. et al., 2022). Moreover, the extra-framework Fe^3+^ in Cu-SSZ-13 could suppress the aggregation of Cu^2+^ at high reaction temperature and promote the transformation of [Cu(OH)]^+^ species to Cu^2+^ during hydrothermal aging, leading to better catalytic performance (Figure 6B) (Yin et al., 2016; Chen M. Y. et al., 2021). The poisoning tolerance could also be increased by the synergistic effects between Fe and Cu species (Zhang et al., 2014). The further base leaching on CuFe-SSZ-13 catalyst could significantly promote the interaction between active metals and zeolite framework, induce the formation of Lewis acid sites, improve redox ability, and effectively promote the generation of Z_2_Cu to significantly accelerate the reaction between the adsorbed NO_*x*_ species with NH_3_, consequently enhancing the SCR performance (Wu et al., 2022).

The heterobimetallic FeCu-SSZ-13 zeolite could also be synthesized by a one-pot strategy (Yue et al., 2020; Wan et al., 2021). The small crystallize size, large specific surface area and pore volume, low acid strength, and abundant isolated Cu^2+^ and framework Fe^3+^ ions endowed the super-wide reaction temperature window, excellent hydrothermal stability, high H_2_O and SO_2_ tolerance, and good gaseous hourly space velocity flexibility of FeCu-SSZ-13 (Yue et al., 2020). Wang M. X. et al. (2019) found that CuFe-SSZ-13 possessed better SO_2_ resistance compared with Cu-SSZ-13 because Fe^3+^ as a sacrificial component interacts with sulfur oxide to protect Cu^2+^ active sites. A different point of view was put forward in CuFe-SSZ-13 prepared by homogeneous precipitation (Xie et al., 2022). The addition of Fe species did not protect Cu species during SO_2_ treatment obviously but retained many active Fe species, which were helpful for the activation of reactants in the presence of SO_2_, especially the reactivity between NH_3_ adsorption species and NO_*x*_, leading to better SO_2_ resistance than Cu-SSZ-13.

Besides, CuCo-SSZ-13 catalysts were synthesized by introducing Co^2+^ ions before loading Cu ions onto Cu-SSZ-13 to improve the SCR activity (Lee et al., 2021a). The pre-loaded Co^2+^ co-cations could induce the Cu^2+^ ions to exist as more reactive species ZCuOH rather than less active species Z_2_Cu because Co^2+^ ions are known to be selectively located at the 2Al sites. Wang J. G. et al. (2021) reported that the low-temperature SCR activity of Cu-SSZ-13 could be dramatically enhanced by introducing a certain amount of niobium (Nb). Similarly, Nb ions entered into the exchange sites to increase the number of ZCuOH and the formation of Nb=O bonds enhanced the amount of the Lewis acid sites and the Brønsted acid sites, promoting the NH_3_ adsorption capacity. Furthermore, Nb modified Cu-SSZ-13 catalyst could adsorb more NO_*x*_ species, which reacted with NH_4_^+^ adsorbed on the Brønsted acid sites through the Langmuir–Hinshelwood (L-H) route, and the coordinated NH_3_ on Lewis acid sites species played main roles as the main active intermediates at low temperature. There is also a study found that Zn could be introduced into the Cu-SSZ-13 catalyst to stabilize Cu^2+^ and thus improve the hydrothermal stability (Xu et al., 2020). Experimental results and DFT calculations confirmed that the introduced Zn could not only help optimize and disperse the isolated Cu^2+^ species, providing good SCR activity, but also form [Zn−OH]-Z, [Cu−O−Zn]-Z, and [Zn−O−Zn]-Z complexes, which were much more stable than the mono-Cu species (ZCuOH) under hydrothermal aging. These Zn-containing species served as anchors to stabilize the SSZ-13 framework and prevent the Cu^2+^ ions from migrating during hydrothermal aging.

### 3.3 Rare earth metal ions

Recently, several rare earth-doped Cu-SSZ-13 catalysts have been widely reported due to their easy operability, outstanding NO_*x*_ removal efficiency, and desired improvement in hydrothermal stability (Table 3) (Liu et al., 2015; Zhao Z. C. et al., 2019; Zhang S. T. et al., 2020). Cerium (Ce) as a modifier has been widely used in SCR catalysts due to its easy redox cycle between +3 and +4 valence states (Tang et al., 2016; Jiang L. et al., 2020). Wang et al. (2016) reported Ce-stabilized Cu-SSZ-13 catalysts, in which Ce exchanged ions stabilized the Cu active centers, partially preventing dealumination of the SSZ-13 framework. In addition, the existence of Ce^3+^ created a charge imbalanced environment, leading to increased oxygen vacancy, improving the activation of surface oxide species, so as to increasing the low-temperature activity. Thereafter, Usui et al. (2018) reported the Ce-incorporated Cu-SSZ-13 exhibited excellent hydrothermal stability since Ce located in the CHA cage of SSZ-13 could fill the defect sites, reduce the number of available attacks positions and stabilize the zeolite structure (Figure 6C). The introduced Ce could change the location and coordination environment of Cu^2+^ ions and participate in the reaction process, which was beneficial for the low-temperature SCR activity (Wang Y. et al., 2021). In the case of one-pot synthesized Cu-SSZ-13, the catalytic activity and sulfur resistance could be enhanced by modifying with Ce using the IE method (Li et al., 2019). The improved redox capacity by doping with Ce and the synergistic effect between Cu and Ce species which enhanced the adsorption of reactants contribute to the excellent SCR performance.

**TABLE 3:** Summary of the Cu-SSZ-13 catalysts doped with rare earth metal ions.

Introduced species	Cu content (wt%)	Doped ions	Dopant content (wt%)	Active temperature window for fresh samples (°C) (NO_*x*_ conversion >90%)	Active temperature window for hydrothermal aged samples (°C) (NO_*x*_ conversion >90%)	References
Rare earth metal ions	2.8	Y	1.3	190–550	190–550	Zhao et al. (2019c)
	1.14	Ce+Fe	0.82 + 3.48	200–550	--	Liu et al. (2015)
	2.2	Ce	1.6	190–550	190–450	Zhao et al. (2019c)
	5.65	Ce	0.93	230–600	230–500	Wang et al. (2016)
	3.6	Ce	0.3	200–500	--	Usui et al. (2018)
	1.83 (CuO)	Ce	120 (ppm, CeO_2_)	200–500	250–450	Wang et al. (2021d)
	5.81	Ce	2.40	150–500	--	Li et al. (2019)
	3.4	La	1.8	190–550	200–450	Zhao et al. (2019c)
	3.84	Ce+La	0.78 + 0.75	180–390	210–270	Chen et al. (2020b)
	3.4	Sm	2.3	190–600	190–450	Zhao et al. (2019c)
	2.39	Sm	0.2	200–550	225–350	Chen et al. (2022a)

Similar enhanced SCR performance has been reported by the addition of yttrium (Y) cations in the Al-rich Cu-SSZ-13 (Zhao Z. C. et al., 2019), furthermore the DFT calculation confirmed that Y^3+^ and Cu^2+^ ions entered the SSZ-13 zeolite to form coordination bonds with the framework O to stabilize the framework Al, consequently enhancing the hydrothermal stability of Cu-SSZ-13 catalyst (Li et al., 2020). Besides the introduction of Ce and Y, Wang Y. J. et al. (2020) also reported that samarium (Sm)-incorporated Al-rich Cu-SSZ-13 (Si/Al = 3.6) showed better hydrothermal stability than the pristine Cu-SSZ-13. The introduced Sm cations were hypothesized to locate at IE sites in CHA cages due to their large ionic radius, which contributed to the inhibition of the dealumination of the zeolites. After hydrothermal aging, Sm-modified Cu-SSZ-13 maintained high crystallinity, large amounts of acid sites and active Cu species to provide superior SCR activity compared to Cu-SSZ-13. Very recently, the incorporation of Sm ions into Cu-SSZ-13 zeolites has been reported for superior SCR performance (Chen M. Y. et al., 2022). The Sm ions are found to occupy the 6 MR of SSZ-13, which facilitated the formation of more active ZCuOH ions at 8MRs, simultaneously the electron transfer from Sm^3+^ to ZCuOH ions not only promoted the activity of ZCuOH ions but also inhibited the transformation of ZCuOH ions into CuO_*x*_ species, thus enhancing the SCR performance of Cu-SSZ-13 catalysts. Although lanthanum (La) doping was demonstrated to decrease the amount of framework Al and Cu active sites of Cu/SSZ-13 after hydrothermally aging (Deng et al., 2021), the co-introduction of Ce^4+^ and La^3+^ ions could effectively regulate the Cu^2+^ ions to migrate from 8 MR to more active 6MRs, endowing Cu-Ce-La-SSZ-13 with excellent low-temperature SCR activity (Chen et al., 2020a).

### 3.4 Metal oxides

Based on the above discussion, several options have been proposed to improve the SCR activity of Cu-SSZ-13 catalysts. An alternative is to add a second phase (metal oxide) to Cu-SSZ-13 to help improve low-temperature activity except for co-exchanging Cu-SSZ-13 with heteroatoms, in which the improvement in low-temperature SCR performance is limited since the co-cations appear to enhance Cu redox capacity, but decrease Cu loading by competing for the exchange sites (Table 4) (Andana et al., 2021; 2022). The metal oxide in Cu-SSZ-13 zeolite-metal oxide composite catalyst carried out the oxidative function to partially convert NO to NO_2_ to enable the fast SCR reaction over the catalyst.

**TABLE 4:** Summary of the Cu-SSZ-13 catalysts composited with metal oxides.

Composited species	Cu content (wt%)	Metal oxides	Dopant content (wt%)	Active temperature window for fresh samples (°C) (NO_*x*_ conversion >90%)	Active temperature window for hydrothermal aged samples (°C) (NO_*x*_ conversion >90%)	References
Metal oxides	1.4	CeMnO_*x*_	1:3 (mass ratio)	200–380	--	Andana et al. (2022)
	--	MnO_*x*_-CeO_2_	6:4:90 (MnO_*x*_:CeO_2_:Cu-SSZ-13, mole ratio)	150–450	--	Liu et al. (2017)
	0.87 (surface at%)	CeWTi oxides	1:4 (mass ratio)	250–500	250–450	Zhao et al. (2021b)
	3.4	CeO_2_-SnO_2_	1:4 (mass ratio)	200–400	250–550 (SO_2_ aged)	Martinovic et al. (2021)
	2.2	ZrO_2_	10.0	200–500 (de-greened)	200–500	Peng et al. (2020)
	2.0	ZnTi_10_O_*x*_	1:5 (mass ratio)	180–500	180–500	Yu et al. (2020)

Among the various metal oxides, MnO_*x*_-CeO_2_ catalyst has been studied extensively due to its nontoxicity and outstanding low-temperature activity, therefore it should be an ideal candidate for improving the low-temperature activity of Cu-SSZ-13 catalyst (Qi et al., 2004). Liu et al. (2017) supported MnO_*x*_-CeO_2_ on Cu-SSZ-13 samples with an impregnation method. The sample had excellent NO_*x*_ conversions above 90% from 125 to 450 °C and good H_2_O and SO_2_ resistances. Recently, the reaction pathway of MnO_*x*_-CeO_2_-Cu-SSZ-13 composite catalyst was revealed, in which the oxide-derived nitrites/nitrite-precursors (e.g., HONO, N_2_O_3_) reacted with Cu-SSZ-13 stored NH_3_ species to generate NH_4_NO_2_ intermediate whose decomposition is almost barrierless assisted by the Brønsted-acid site (Figure 6D) (Andana et al., 2022). Furthermore, this composite catalyst could circumvent the challenge of NH_4_NO_3_ deposits inhibiting low-temperature SCR on Cu-SSZ-13.

Other Ce-based oxides were also developed as the metal oxide component of the composite catalyst (Zhao W. Y. et al., 2021; Martinovic et al., 2021). The Cu-SSZ-13/CeWTi composite catalysts formed a micro-mesoporous structure, and the improved crystal structure of SSZ-13 and high specific surface area and pore volume are conducive to enhancing the low-temperature SCR activity (Zhao W. Y. et al., 2021). The combination of micropores and mesopores produced more Ce^4+^ ions, surface chemisorption oxygen species as well as acid sites to enhance the low-temperature SCR performance and hydrothermal stability of the catalyst. The Cu-SSZ-13 mixed with CeO_2_-SnO_2_ to form a composite catalyst *via* a solid-state synthesis, which was resistant to hydrocarbon poisoning of the SCR reaction due to the interaction between Cu, CeO_2_-SnO_2_, and protonic sites (Martinovic et al., 2021).

There are also studies focusing on reducing the adverse effects of hydrothermal aging on Cu-SSZ-13 catalysts by introducing zirconium oxide (ZrO_2_) (Peng et al., 2020). A strong interaction was achieved by the highly dispersed ZrO_2_ on Cu-SSZ-13, and a Cu-stabilized *t*-Zr O _2_ phase formed, which greatly hindered the non-selective NH_3_ oxidation due to CuO_*x*_ (Figure 6E). Therefore, the high-temperature SCR activity was enhanced without reducing the SCR activity at low temperatures. Yu et al. (2020) found that some titanium (Ti)-based metal oxides (XTi_10_O_*x*_, X = Mn, Co, Ni, Zn), which can be mixed with Cu-SSZ-13 zeolite to form hybrid catalysts, can serve as a sacrificial component to prevent Cu^2+^ site poisoning. Notably, the ZnTi_10_O_*x*_-Cu-SSZ-13 catalyst showed a significantly higher NO conversion than in Cu-SSZ-13 after poisoning with SO_2_, in which more ZnSO_4_ species were generated, sparing the Cu^2+^ active sites from sulfur deactivation.

## 4 Regulating the morphology

The morphology of the Cu-SSZ-13 catalyst may also impart effects on the performance and stability of the catalysts, consequently regulating the morphology is also an effective way to improve the performance of Cu-SSZ-13 catalysts (Table 5). It includes modulating the crystalline size of zeolites and introducing mesoporous structures into the microporous zeolites to reduce the diffusion limitation of reactants and product molecules, thus improving the mass transfer rate and catalytic performance of zeolites; introducing hydrophobic surface on the zeolite to alleviate the attack from water, suppressing the dealumination of the SSZ-13 zeolite, thus improving the hydrothermal stability (Hartmann et al., 2016; Takata et al., 2016; Gallego et al., 2018; Wardani et al., 2018; Sun et al., 2020).

**TABLE 5:** Typical Cu-SSZ-13 catalysts with different morphologies reported in the literatures.

Morphology	Method	Etching agent/Template	Specific surface area (m^2^/g)	Pore/Partical size (nm)	Active temperature window (°C) (NO_*x*_ conversion >90%)	References
Sub-micron particle size 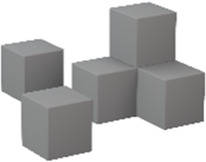	Hydrothermal (Changing the gel composition and hydrothermal aging time)	TMAdaOH	--	450	200–500	Prodinger et al. (2017)
		TMAdaOH	575	216–288	175–450	Palčić et al. (2020)
	Two-stage synthesis	TMAdaOH	--	50–150	220–450	Peng et al. (2018)
	Hydrothermal (Adding seed crystals)	1-Adamantanamine	843	2,300	240–390	Chen et al. (2021b)
	FAU interzeolite	TMAdaOH	858	120	175–450	Liang et al. (2020)
Hierarchical pores 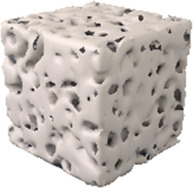 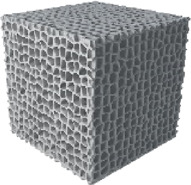	Desilication	NaOH	597	1.0–3.0	250–450	Oord et al. (2017)
	Dual-template	TMAdaOH+C_16_MP	537.5	3.6	240–450	Liu et al. (2020b)
		TMAdaOH+BC_ph-10-6-6_	424	5.5	175–500	Peng et al. (2021)
	Hard template	Carbon black	664	25	180–500	Liang et al. (2021)

### 4.1 Modulating the crystalline size of zeolites

Sub-micron Cu-SSZ-13 was shown to be an effective and stable catalyst for SCR reaction (Prodinger et al., 2017). However, the catalytic performance and hydrothermal stability of the Cu-SSZ-13 catalyst are independent of the particle size. Conversely, Palčić and coworkers (2020) found that decreasing the size of zeolite crystals could improve the efficiency of the IE process to generate more Cu^2+^ sites in Cu-SSZ-13. Cu-SSZ-13 catalyst with particle size ∼300 nm possessed more Cu^2+^ in the 6 MR and less CuO species leading to its higher SCR activity compared to other catalysts with smaller particle sizes. Moreover, the hydrothermal stability of the Cu-SSZ-13 catalyst was also affected by the nano-size effect. Peng et al. (2018) developed a two-stage synthetic method conducted at low (95°C) and high (210°C) temperatures sequentially to prepare nanosized SSZ-13 zeolite. The obtained nanosized Cu-SSZ-13 zeolite exhibited enhanced hydrothermal stability due to the structural healing of SSZ-13 by the high-temperature treatment.

There were still some debates about the nano-size effect on the poisoning resistance of Cu-SSZ-13. Compared with nanosized counterparts, Cu/SSZ-13 with a crystal size of 2.3 µm exhibited excellent resistance to Na poisoning (Chen Z. X. et al., 2021). The large crystal size facilitated the distribution of acidic sites and the Cu ions distributed in the deep layer have strong redox stability and are not easily solvated by NH_3_ or H_2_O, and are less likely to be replaced by Na to generate CuO. However, a different perspective was offered by Liang and colleagues, in which the SCR activity as well as the SO_2_ and H_2_O resistance over the nano-sized Cu-SSZ-13 outperformed the conventional Cu-SSZ-13 mainly due to the much shorter diffusion path (Liang et al., 2020).

### 4.2 Fabricating hierarchical pores

Since SSZ-13 is a small pore 8 MR structure with a radius of 3.8 Å, it also causes problems such as diffusion limitations that limit the physical transport of reactants to the active sites (Liu et al., 2020a). Indeed, the internal diffusion limit plays a key role in the NH_3_-SCR performance of Cu-SSZ-13, especially at low temperatures (Gao et al., 2013b). Therefore, the addition of mesopores (2–50 nm) could eliminate diffusion limitations and promote the accessibility of reactants to the active sites in the zeolite (Hartmann et al., 2016; Sun et al., 2020). In recent years, hierarchical zeolites containing both micro and meso-/macrospores have been proved to improve catalytic performance efficiently by accelerating the mass transportation of the reactants and products. Some Cu-SSZ-13 catalysts with hierarchical pores reported in the literatures are listed in Table 5.

Mesoporous Cu-SSZ-13 was first created by desilicating using NaOH leaching, and it became more active in SCR reaction, especially in the low-temperature region (Oord et al., 2017). This promoted activity could be attributed to decreasing the pore diffusion limitations because of the introduction of mesopores on the outside of the zeolite crystals. In addition to the post-treatment, direct synthesis is the main synthesis method of hierarchical zeolites. Liu et al. (2020b) developed a dual-template strategy combining TMAdaOH as the structure-directing agent with C_16_H_33_-[N^+^-methylpiperidine] (C_16_MP) as the mesoporogen to build the pore-controllable hierarchical Cu-SSZ-13 catalyst. The interaction between micropores and mesopores improved the adsorption of NO and NH_3_ and the mesopores maintained the high-temperature activity due to their inhibition of NH_3_ oxidation. Furthermore, the highly crystalline hierarchical porous Cu-SSZ-13 zeolite also exhibited improved SO_2_ tolerance due to the mesopores alleviating the accumulation of sulfates (NH_4_HSO_4_ (NH_4_)_2_SO_4_, *etc.*) (Peng et al., 2021)., Notably, the highly crystalline hierarchical porous Cu-SSZ-13 could be synthesized by adding carbon black as a hard template (Liang et al., 2021). The obtained Cu-SSZ-13 catalyst with ordered mesopores exhibited superior performance, especially the low-temperature deNO_*x*_ performance compared with the traditional Cu-SSZ-13 without mesopores. More importantly, Cu-SSZ-13 catalyst with ordered mesopores displayed superior hydrothermal stability, as well as water and sulfur resistance because the ordered mesopores could prevent the formation of NH_4_NO_3_ and promote the decomposition of sulfates.

### 4.3 Constructing the core-shell structures

**FIGURE 7:**
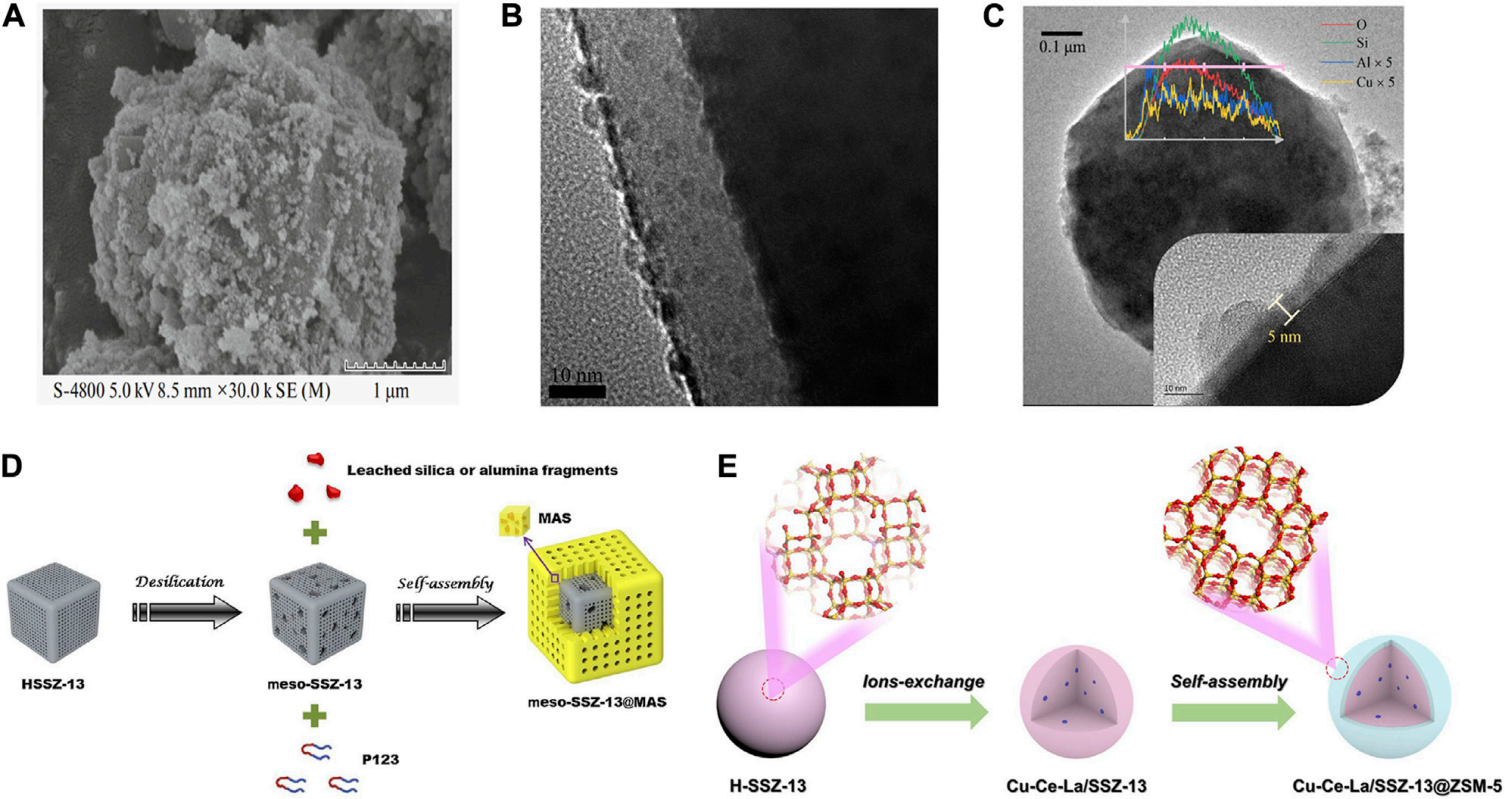
Core-shell structured Cu-SSZ-13 catalysts. **(A)** SEM image of Cu-SSZ-13@CeO_2_ catalyst. Reproduced with permission Shi et al. (2020), Copyright 2020, Springer Publishers. **(B)** TEM image of SiO_2_ nanolayer coating on the Cu-SSZ-13 catalyst. Reproduced with permission Tian et al. (2021), Copyright 2021, Elsevier Publishers. **(C)** TEM images and EDS line scanning of Al-Cu-SSZ-13. Reproduced with permission Ma et al. (2022), Copyright 2022, Elsevier Publishers. **(D)** Schematic representation of the preparation of meso-SSZ-13@MAS composite. Reproduced with permission Zhang et al. (2016a), Copyright 2016, Elsevier Publishers. **(E)** Schematic representation of the preparation of the Cu-Ce-La/SSZ-13@ZSM-5 core-shell catalyst. Reproduced with permission Chen et al. (2020b), Copyright 2020, Elsevier Publishers.

The core-shell structure for improving the SCR performance has been employed in Cu-SSZ-13 catalysts. A series of Cu-SSZ-13@CeO_2_ catalysts with surface modification with CeO_2_ was prepared by the modified self-resemble method based on the one-pot synthesized Cu-SSZ-13 catalyst (Figure 7A) (Shi et al., 2020). The low-temperature SCR activity and the SO_2_+H_2_O tolerance of Cu-SSZ-13@CeO_2_ were found to enhance markedly compared with Cu-SSZ-13. The improved redox property as well as the increased adsorption of NH_3_ and NO_*x*_ species by the CeO_2_ modification were crucial for the enhanced SCR performance of Cu-SSZ-13@CeO_2_.

Modifying the surface of Cu-SSZ-13 catalysts by introducing a hydrophobic surface, protecting shell, or metal oxides could prevent the water molecules from attacking the surface of a zeolite catalyst, and eventually improve the hydrothermal stability of catalysts. Tian et al. (2021) reported a new strategy to improve the high-temperature hydrothermal stability of Cu-SSZ-13 by coating the surface with a SiO_2_ nanolayer using the atomic layer deposition (ALD) method (Figure 7B). The deposited thin and uniform SiO_2_ nanolayer scarcely affects the SCR activity of Cu-SSZ-13 but improves the high-temperature hydrothermal aging resistance by maintaining the integrity of the frameworks of Cu-SSZ-13 and slowing down the accumulation of Cu species as well as stabilizing the position of Cu^2+^ ions to prevent the formation of CuO clusters during hydrothermal aging.

The same effect can also be achieved by coating the surface of the catalyst with a thin Al_2_O_3_ layer by a chemical liquid deposition (CLD) method (Figure 7C) (Ma et al., 2022). The high-temperature SCR activity of the Al-modified Cu-SSZ-13 was maintained upon 750–800°C hydrothermal aging, and the over-oxidation of NH_3_ was largely inhibited by the absence of CuO-like species due to the formation of CuAlO_2_-like species upon Al modification during hydrothermal aging. In addition, a core-shell structured Cu-SSZ-13 fabricated by a mesoporous aluminosilicate (MAS) shell coating on the mesopore-containing SSZ-13 core could simultaneously solve three major problems existing in the previous Cu-SSZ-13, i.e., low-temperature activity, hydrothermal stability, and propene poisoning resistance (Figure 7D) (Zhang et al., 2016a). The increase of SCR activity across the entire temperature range was attributed to increasing the number of active Cu species (isolated Cu^2+^ ions) and fewer pore diffusion limitations. The MAS shell prevented the dealumination of the SSZ-13 core effectively, hence enhancing the hydrothermal stability of the catalyst.

A core-shell structure Cu-Ce-La/SSZ-13@ZSM-5 catalyst with a Cu-Ce-La/SSZ-13 core and a ZSM-5 shell was synthesized by a self-assembly method (Figure 7E) (Chen et al., 2020b). In comparison with Cu-Ce-La/SSZ-13, Cu-Ce-La/SSZ-13@ZSM-5 with appropriate shell thickness presents better SCR activity and hydrothermal stability. Part of the metal ions was migrated and redistributed during the assembly of the ZSM-5 shell, resulting in the transformation of ZCuOH to Z_2_Cu species and the functionalization of the shell phase to be conducive to the adsorption and activation of NH_3_ for promoting SCR activity. Meanwhile, the hydrophobic shell effectively improves the hydrothermal stability and H_2_O resistance of core-shell Cu-Ce-La/SSZ-13@ZSM-5.

## 5 Summary and outlook

In this review, the research progress in optimizing the SCR performance of Cu-SSZ-13 for deNO_*x*_ has been summarized. Considering the problems existed in practical application, the strategies such as modifying the Cu active sites, introducing the heteroatoms or metal oxides, and regulating the morphology are thoroughly discussed for improving the low-temperature activity, hydrothermal stability, and poisoning resistance. Despite the widespread investigation and efficient utilization as commercial SCR catalysts, Cu-SSZ-13 catalysts still have some challenges and remaining issues listed as follows.

1) The current synthesis methods and the corresponding Cu-SSZ-13 catalysts often suffer from complex procedures, relatively high costs, low Cu loadings, and inferior deNO_*x*_ efficiency, which would inhibit their large-scale production and industrial application. Based on the development of data analysis and predictions, emerging techniques such as high throughput screening, machine learning, or a combination of both will be challengeable tasks in searching for the new synthesis methods, technological parameter as well as optimal performance of Cu-SSZ-13 catalysts.
2) Although many efforts, especially involved in introducing heteroatoms, have been devoted to optimize the SCR performance of Cu-SSZ-13 catalysts, the investigation of improved mechanisms and the reaction pathway should be strengthened. The atomic-level understanding of the electronic effect and steric effect between the doped atoms and Cu^2+^ ions as well as the effect of doped atoms on zeolite framework and active sites are worthy of study by suitable experiments combined with auxiliary calculations.
3) Besides the metal oxides/Cu-SSZ-13 composite catalysts mentioned in this review, development of other composite catalysts composed with different elemental composition and pore structure for the NH_3_-SCR reaction should be pursued. The combination of the advantages of the high-performance SCR catalysts are urgently needed, such as the combination of Cu-SSZ-13 with transition metal-based oxides, rare earth-based oxides, Fe-SSZ-13, and Cu-based small-pore zeolites with various structures (AEI, LTA, *etc.*). The composing form, proportion and synergistic effects of the two components in these composite catalysts should also be investigated deeply and thoroughly. Furthermore, the small-pore intergrown zeolites (e.g., AFX/CHA, CHA/AEI) have drawn much attention of researchers, which offer distinctive properties compared with pure zeolite phases. Therefore, Cu-SSZ-13 composite catalysts as well as Cu-CHA intergrown zeolites might be the attractive candidates for future SCR catalysts.
